# Supplementary material for: In Vitro Antifungal Activity of Chimeric Peptides Derived from Bovine Lactoferricin and Buforin II against Cryptococcus neoformans var. grubii
Source: Antibiotics (Basel). 2022 Dec 15;11(12):1819. doi: 10.3390/antibiotics11121819 (PMC9774238; doi:10.3390/antibiotics11121819)

In Vitro Antifungal Activity of Chimeric Peptides Derived from Bovine Lactoferricin and Buforin II against *Cryptococcus neoformans* var. *grubii*

Silvia Katherine Carvajal [1], Yerly Vargas-Casanova [1], Héctor Manuel Pineda-Castañeda [2], Javier Eduardo García-Castañeda [3], Zuly Jenny Rivera-Monroy [2] and Claudia Marcela Parra-Giraldo [1,*]

Table S1. Strains used in the study.

	**Code**		**Description**
**Reference strain**	H99		Parental strain from the H. Madhani 2007 collection: CM018 deposited at FGSC
		**Gen**	**Function**
**Mutants**	CNAG_04693	*sin1*	Target of rapamycin complex 2 subunit
	CNAG_01580	*scp1*	Sterol regulatory element-binding protein cleavage-activating protein
	CNAG_04804	*sre1*	Sterol regulatory element-binding protein
	CNAG_06925	-	Arsenical-resistance protein
	CNAG_02050	*qdr1*	Putative MFS multidrug transporter
	CNAG_02430	-	ABC transporter ABCC.6
	CNAG_06348	*pdr5-3*	ABC transporter PMR5
	CNAG_00730	*afr1*	ATP-binding cassette transporter
	CNAG_05150	-	ATP-binding cassette transporter
	CNAG_02341	-	Cyclin binding protein
	CNAG_02915	*pdk1*	AGC/PDK1 protein kinase
**Clinical isolates**	2807		Isolated from HIV positive patient
	3279		N/I
	2643		Isolated from HIV positive patient

(-) Mutants that do not have an associated gene name according to FungiDB.
N/I: no information.

[K]-BFII (32-35)Pal	µg/mL	Inhibition (%)						
	200	7,2	0,0	0,0	0,3	0,0	0,0	0,0
	100	8,2	0,0	1,6	2,5	0,0	0,0	0,0
	50	10,0	0,0	0,0	0,0	0,0	0,0	0,0
	25	11,9	3,1	0,6	1,9	0,0	0,0	0,3
	12.5	9,4	0,9	0,0	0,0	0,0	0,0	0,0
	6.25	6,9	1,3	0,0	3,4	0,0	0,0	0,0
C+	0		0,0	0,0	0,0	0,0	0,0	0,0
			3.125	6.25	12.5	25	50	100
			LfcinB (20-25)					

[K]-LfcinB (20-25)	µg/mL	Inhibition (%)						
	200	0,0	0,0	0,0	0,0	0,0	100,0	100,0
	100	0,0	0,0	0,0	0,0	0,0	0,4	99,6
	50	0,0	0,0	0,0	0,0	0,0	0,0	92,1
	25	0,0	0,0	0,0	0,0	0,0	0,0	40,9
	12.5	0,0	0,0	0,0	0,0	0,0	0,0	41,9
	6.25	0,0	0,0	0,0	0,0	0,0	0,0	14,7
C+	0		0,0	0,0	0,0	0,0	0,0	91,8
			3.125	6.25	12.5	25	50	100
			BFII (32-35)pal					

Figure S1. Effect of the physical combination between: **(a)** [K]-BFII (32-35)Pal (KLLKKLLK) and LfcinB (20-25) (RRWQWR); **(b)** [K]-LfcinB (20-25) (KKWQWK) and BFII (32-35)Pal (RLLRRLLR). C+: growth control.

Figure S2. Chromatographic profile and MS spectra of peptides evaluated. LfcinB [20-25] (A), BFII [32-35]Pal (B), C5 (C) and C6 (D) (18).

A LfcinB (20-25): RRWQWR

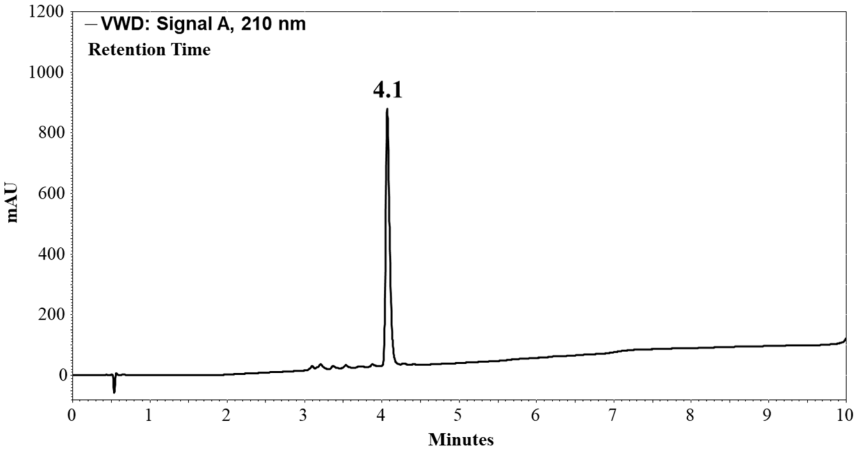

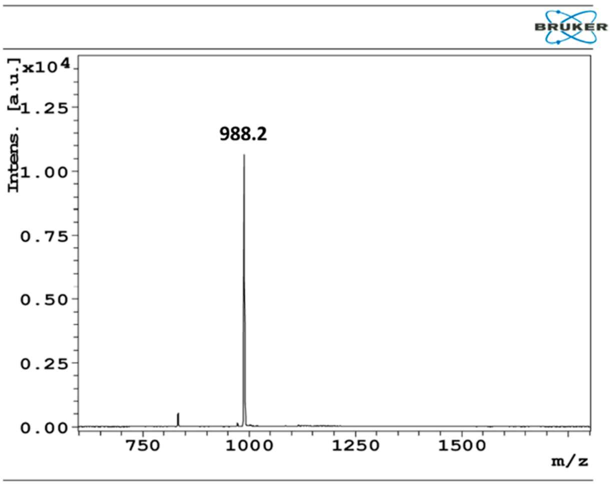

B BFII (32-35): RLLRRLLR

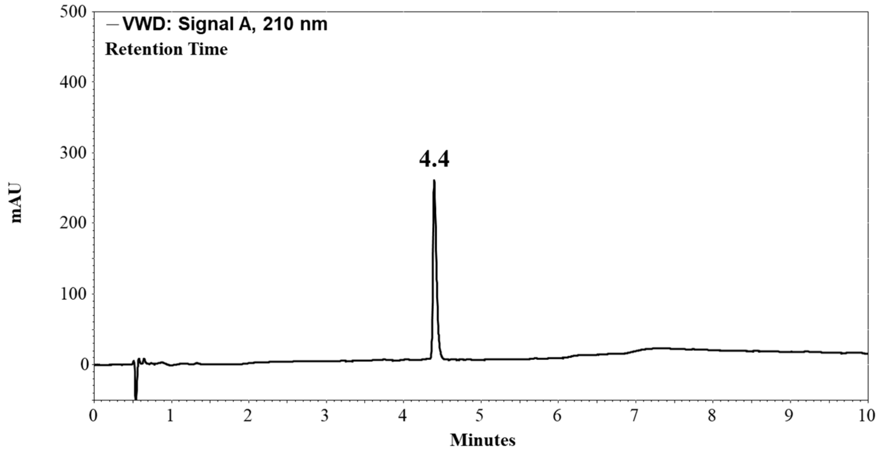

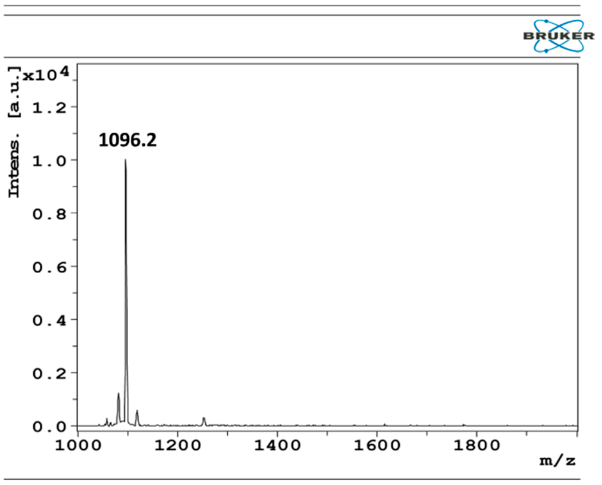

C C5: RRWQWR-Ahx-KLLKKLLK

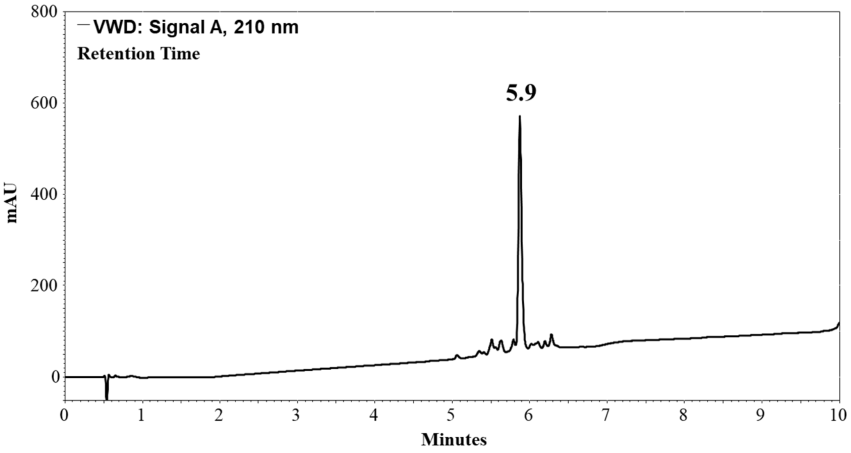

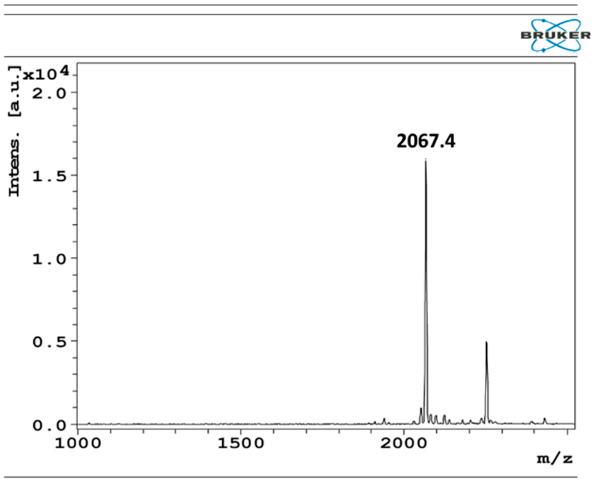

D **C6: KKWQWK-Ahx-RLLRRLLR**

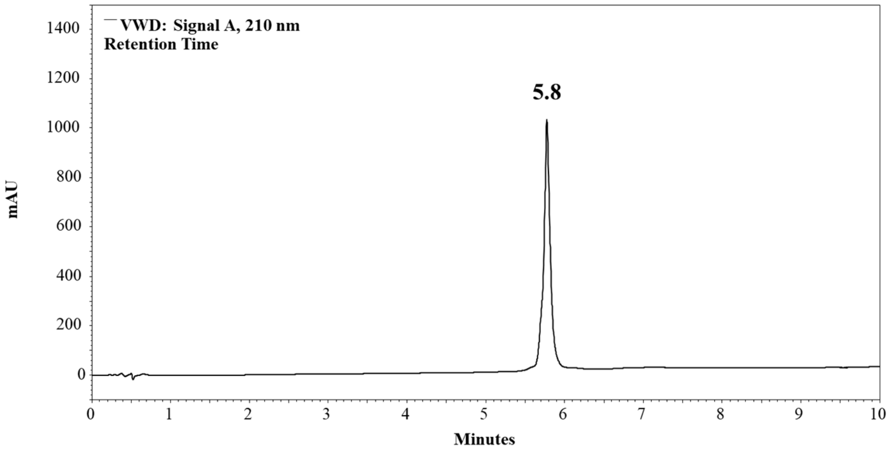

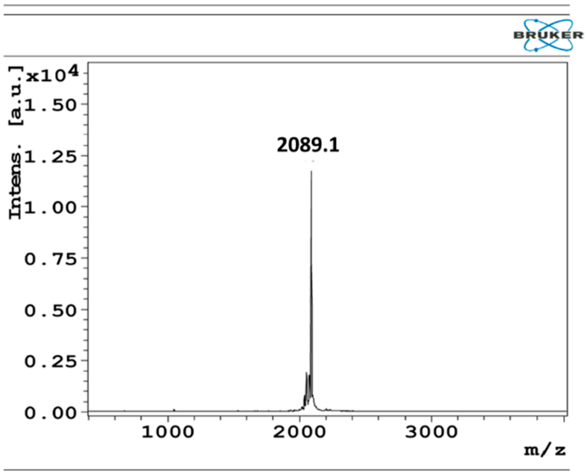

Supplement: Supplementary file 1 [file antibiotics-11-01819-s001.zip › antibiotics-2058293-supplementary.pdf]
